# Land-based drip-irrigated culture of *Ulva compressa*: The effect of culture platform design and nutrient concentration on biomass production and protein content

**DOI:** 10.1371/journal.pone.0199287

**Published:** 2018-06-27

**Authors:** Wilson Mendoza, Dominick Mendola, Jang Kim, Charles Yarish, Alyssa Velloze, B. Greg Mitchell

**Affiliations:** 1 Scripps Institution of Oceanography, Integrative Oceanography Division, University of California, San Diego, La Jolla, California, United States of America; 2 Department of Ecology & Evolutionary Biology, University of Connecticut, Stamford, Connecticut, United States of America; The Education University of Hong Kong, HONG KONG

## Abstract

This work developed a laboratory prototype methodology for cost-effective, water-sparing drip-irrigation of seaweeds, as a model for larger-scale, on-land commercial units, which we envision as semi-automated, inexpensive polyethylene sheet-covered bow-framed greenhouses with sloping plastic covered floors, water-collecting sumps, and pumped recycling of culture media into overhead low-pressure drip emitters. Water droplets form on the continually wetted interior plastic surfaces of these types of greenhouses scattering incoming solar radiation to illuminate around and within the vertically-stacked culture platforms. Concentrated media formulations applied through foliar application optimize nutrient uptake by the seaweeds to improve growth and protein content of the cultured biomass. An additional attribute is that seaweed growth can be accelerated by addition of anthropogenic CO_2_-containing industrial flue gases piped into the head-space of the greenhouse to reuse and recycle CO_2_ into useful algal biomass. This demonstration tested three different drip culture platform designs (horizontal, vertical and slanted) and four increasing fertilizer media concentrations (in seawater) for growth, areal productivity, and thallus protein content of wild-collected *Ulva compressa* biomass, against fully-submerged controls. Cool White fluorescent lights provided 150–200 μmol photon m^-2^ s^-1^ illumination on a 12/12 hr day/night cycle. Interactive effects we tested using a four-level single factorial randomized block framework (p<0.05). Growth rates and biomass of the drip irrigation designs were 3–9% day^-1^ and 5–18 g m^-2^ day^-1^ (d.w.) respectively, whereas the fully-submerged control group grew better at 8–11% per day with of 20–30 g m^-2^ day^-1^, indicating further optimization of the drip irrigation methodology is needed to improve growth and biomass production. Results demonstrated that protein content of *Ulva* biomass grown using the vertically-oriented drip culture platform and 2x fertilizer concentrations (42:16:36 N:P:K) was 27% d.w., approximating the similarly-fertilized control group. The drip methodology was found to significantly improve gas and nutrient mass transfer through the seaweed thalli, and overall, the labor- and-energy-saving methodology would use a calculated 20% of the seawater required for conventional on-land tank-based tumble culture.

## Introduction

Reports by The Food and Agriculture Organization of the UN (FAO) project a doubling in demand for seafood in the 21st century, with most of the increased demand to be met by aquaculture [1–5]. In Asia and other parts of the world, seaweed has been a cost-effective food commodity cultured using a traditional long-line method in coastal seas [1, 6]. However, much of the future demand for aquacultured products will be for protein-rich fish and/or shrimps that currently are fed almost exclusively on fishmeal-containing manufactured aquafeeds [7, 8], which is viewed as an environmentally, ecologically and socially unsustainable practice [4, 9,10].

Currently, obtaining permits for practicing aquaculture (mariculture) in near-shore coastal regions of the continental United States is a very lengthy and expensive process layered with local, state and national regulations [11, 12]. In contrast, mass culture of seaweeds using water-sparing, recycling methods in land-based greenhouses would be much easier to permit, given that the practice resembles standard agriculture/ horticulture practiced in greenhouses [13–16]. In addition, modern land-based seaweed cultivation in ponds and/or in tumble-type tanks requires copious amounts of continuously renewed seawater, and continuous agitation of culture waters, consuming large amounts of energy (mostly of fossil origin), often exceeding the energy content of the marine biomass produced [17,18]. Marine seaweeds can be consistently cultured to produce thallus protein content in the range of 35% on a dry weight basis [13, 17, 19], with the highest reported values approaching 50% d.w. [20]. With these appreciable levels of high quality marine protein becoming increasingly available from seaweed cultivation, the aquaculture feed industry is seeing a gradual shift in high quality protein sourcing from high-priced, and unsustainably-produced fishmeals to lower cost marine macrophyte meals, for at least partial replacement in aquafeeds [21] Furthermore, fishmeal and fish oil supplies are diminishing due to heavy fishing pressure to supply the growing need for aquaculture feeds, and costs have been rising, and increasingly, alternative protein sources from plants are being sought and substituted [8]. However, the increased use of terrestrial plant protein such as soy meal impacts food costs and nutrition for humans living in developing nations [4]. In this developing scenario there is little potential for increasing fishmeal supplies for aquafeeds [21], so new aquafeed formulations incorporating marine macroalgal (seaweed) protein to reduce the proportion of fishmeal in feeds are logical alternatives, which can, in the process, increase the sustainability index of the feeds produced [22–26].

The use of recirculating drip irrigation culture methods for growing macroalgae in controlled environments on land is a new and mostly un-tried methodology. Given that the method conserves water and nutrient resources it could enable inland culture of seaweeds in high-yield, partially automated aquafarms. The method can also be used for effluent clean-up and water recycling for fish and shrimp culture in an integrated multi-trophic methodology (IMTA) [27]. In addition, and when practiced in simple plastic film-covered greenhouses, the method allows for day-time introduction of CO_2_-rich flue gases from industrial point sources directly into head-spaces of the plastic-covered greenhouses, providing inexpensive, recycled inorganic carbon to the drip irrigated seaweeds. Moreover, one important advantage also of land-based cultivation, including the drip irrigation methods, is the ease and flexibility of controlling the culture system [28, 29]. This ensures that production meets high quality standards and biosafety for human and animal consumption, as well as for other high value applications such as cosmeceutical or pharmaceutical products. Due to these reasons, there have been significant attempts of land-based seaweed cultivations, especially in western countries [30–34].

*Ulva* spp. (Chlorophyta, “green seaweeds”) are ideal candidates for land-based cultivation for many reasons. Research has shown that *Ulva* species express high growth rates and biomass yields, and provide effective bioremediation benefits [17, 27,35]. As whole dried biomass, they can be a good source of marine protein for inclusion into aquafeeds [23,24]. This seaweed group is dominant within marine littoral zones from around the world, where it grows prolifically and vegetatively in thick, green mats. Some *Ulva* species are known to withstand extreme desiccation at the high inter-tidal zone, even under the direct heat of the sun; and with the next incoming tide they are quickly revived to full turgor and health [36–38]. Interestingly, desiccation has been found to enhance nitrogen uptake rates, tissue nitrogen and protein contents in some intertidal seaweeds [39–41]. Also, seaweeds have the capacity to bio-accumulate not only essential elements but also exhibit a high affinity heavy metals and other toxic substances [42–44]. Although brown algae usually have a higher metal-binding capacity than green algae [45], *Ulva* can take up solubilized metals efficiently due to its high surface-area-to-volume ratio [46].

**Fig 1:**
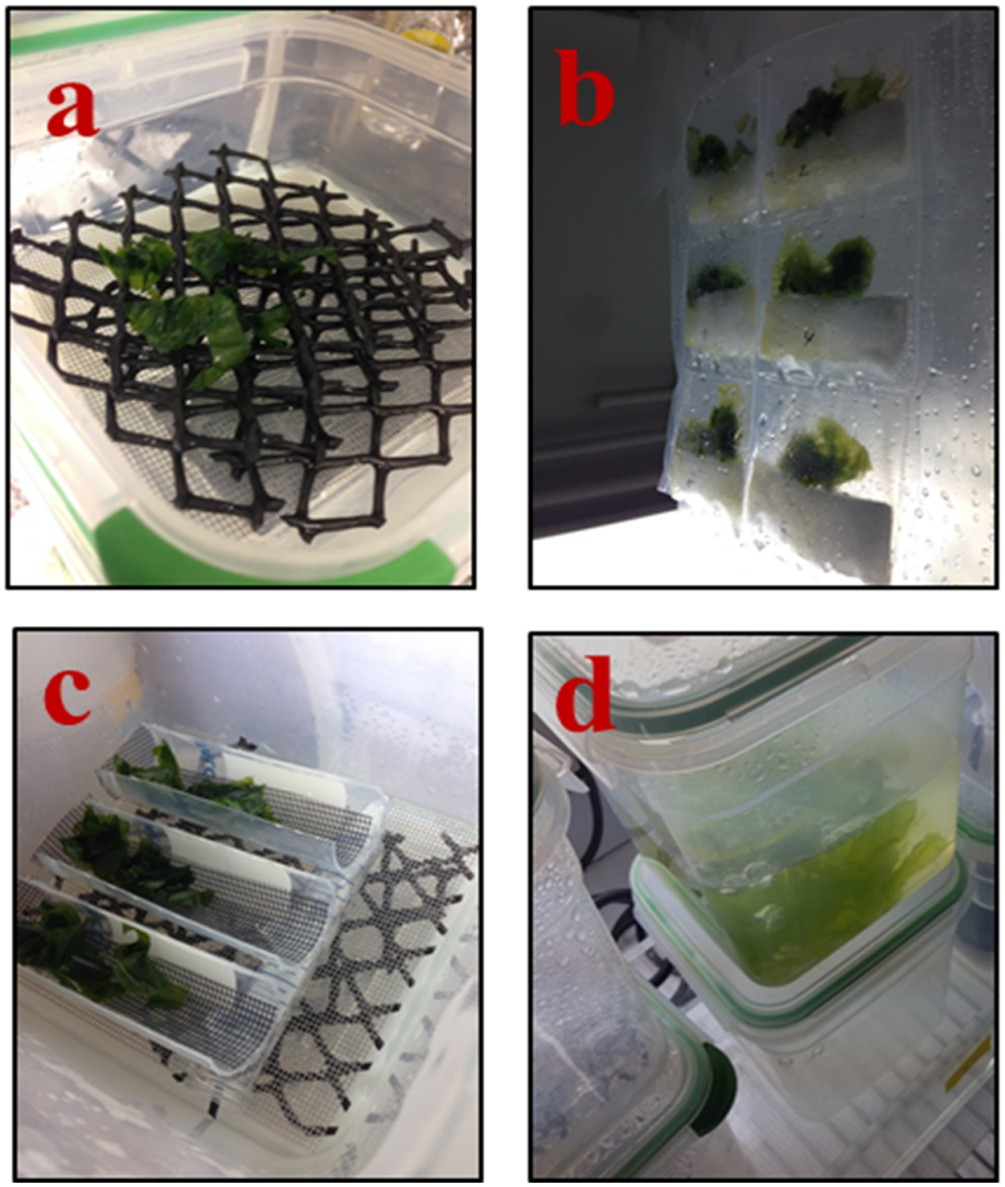
Seaweed cultivation platforms of *Ulva compressa* grown at different JSM (mean ± st.dev., n = 3) concentrations on three different cultivation spray systems for 15 days. **a**) Multi-Level Horizontal Design (MLHD); **b**) Bag-Pocket Vertical Design (BPVD), **c**) Sloped Design (SD), **d**) Submerged (SUB). Except for the submerged, the other three designs were recirculating systems where the recycled water was pumped back into a reservoir that collects the water and drips back into the culture through gravity at a flow rate of ~5 ml s^-1^. Two fluorescent lamps were placed on both sides of the systems set at a 12/12 light/dark cycle. It is a randomized two-factorial design (see S1 Fig for illustration) to test the design and JSM concentration and its interaction effects with growth performance and protein production.

In this study we tested three drip irrigation cultivation platform designs against fully-submerged controls (Fig 1 and S1 Fig), over a range of fertilizer concentrations, to test growth response, protein content, and overall areal biomass productivity, in order to be able to project capital costs and operating expenses (in a follow-on analysis) for hypothetical scale-up of land-based seaweed farms to supply high-protein seaweed meals to aquafeed mills.

## Materials and methods

Three drip irrigation platform designs were evaluated and tested in triplicate. The first was a Multi-Level Horizontal Design (MLHD), three, identical, vertically-stacked and tightly coupled square plastic containers (8” x 8” base x 3” height) were used. The uppermost container served as a media holding reservoir to receive input media via a 4-mm silicone rubber tube attached to a small, submerged aquarium-type pump located in the bottom-most container. The bottom of the upper reservoir was totally drilled with an array of equally-spaced 2 mm-diameter holes on a 5 x 5 mm spacing pattern, and thus served as the drip irrigation provider. The seaweed biomass for testing was contained in the middle container which had a similar hole-drilled bottom covered by a square fine-mesh nylon netting to prevent clogging of the holes by small pieces of seaweed. To commence an experiment, a pre-weighed mass of *Ulva* seaweed thalli was placed into the middle container and the three containers tightly coupled together. The pump was switched on to commence the recirculation of the culture media at a plastic valve-set rate flow rate (approx. 1 ml s^-1^), and the date and time were recorded to begin the experiment.

The second platform design was termed a Bag-Pouch Vertical Design (BPVD), e.g., a vertical-array of three small clear plastic mesh bag-pouches were tied one above the other on a long poly string. The small pouches would hold the seaweed biomass, while culture media was dripped from a reservoir above cascading down the array from pouch-to-pouch to a collection reservoir below. A number of fiberglass wool strips were placed inside the bag-pouch containers to serve for water retention and prevention of mesh clogging, and for physical cushioning for the plants, which were continually being “pelted” by water drips throughout the experiments. The bottom reservoir contained the same type of small aquarium pump attached to a flow-control valve and recirculating tubing as described for the horizontal design.

The third platform design termed a Sloped Design (SD) which was fashioned from 50-ml plastic laboratory culture tubes cut into half-sections and arranged in a horizontal sloping formation. This arrangement allowed the culture media to run through the seaweed pieces held on mesh strips placed into the sloping half-tubes, and then into a bottom collection reservoir where a submerged aquarium pump continuously recirculated the culture media to an upper drip reservoir.

For the submerged control treatments (SUB) the treated seaweed pieces (see section which follows) were free-floating in the treatment container which was continuously recirculated using a small, screen submerged aquarium pump.

Two Philips F40T12/DX Hg-vapor “Cool White” fluorescent light tubes were used on opposite sides of each of the drip-culture modules and control containers, to provide as near as was possible a uniform lighted environment for all culture containers. The light banks were controlled by a timer to provide a 12:12 hr. light/dark cycle, and no overhead lights were left illuminated in the laboratory room throughout the experimental period. Illumination levels around each culture platform were measured using a Biospherical Instruments (San Diego, CA, USA) QSL-100 photosynthetically available radiation (PAR) meter. Some differences in light levels were measured within and between the various culture platforms, encompassing a range of 150–200 -photon m^-2^ s^-1^ illumination measured at the outer surfaces of the culture containers

Air temperatures around the culture arrays were not rigidly controlled other than by the room thermostat. Room air temperatures ranged from 22–24°C throughout the 15-day experimental periods, and culture media temperatures were digitally measured in the collection reservoirs every other day. Salinity of the culture media varied and ranged from 33–35 ppt in any one of the culture platforms throughout the experimental periods.

To fertilize the seaweed masses used in the culture experiments, Jacks Special fertilizer mix was used, (JSM; Jack’s Special^™^ #77440, J.R. Peters, Inc., Allentown, PA, USA; N:P:K = 21:8:18). Fertilizer concentrations were mixed into double-filtered natural seawater, which was freshly-collected as needed from SIO pier’s 60μ-filtered seawater supply line, which draws from La Jolla Bay water at 12-feet deep, 1,000 feet offshore into the Pacific Ocean. In the laboratory, a second filtration was accomplished using a 1 μ woven fiber filter bag. Fertilizer treatments were prepared at 1X, 2X, 4X, and 8X stock concentrations in each media as reported previously by our group [29]; e.g., 1X = 21:8:18 N:P:K; 2X = 42:16:36 N:P:K, etc. We measured dissolved nutrient concentrations in each media treatment before and at the end of each experiment.

The initial biomass weight of Ulva used for each experimental treatment (8 in all, 3 experimental designs x 2 replicates, plus 2x SUB controls) was 12 g. w.w. ± approx. 0.25 g per treatment. Biomass wet weight and (AFDW, pH, Chlorophyll (Chl), light level, nutrients (N and P), and protein content were analyzed for each treatment at the beginning and at the end of the experiments. Total protein content (as % of dry matter weight) was measured using a (CHN) elemental analyzer, and estimated using the nitrogen to protein conversion factor of 5.13 for green seaweeds [47]. Chlorophyll pigments were extracted and quantified using a spectrophotometric analytical method [48]. The seawater media base was autoclaved prior to preparation of the JSM media concentrations (1X, 2X, 4X, and 8X), which contained specific concentrations of NO_3_^-^, NH_4_^+^, DIN and PO_4_^-3^ as shown in Table 1. Between runs, the containers were soaked in, then cleaned with hydrogen peroxide, followed with lab detergent in warm tap water, and rinsed with copious amount of running tap water, followed by rinsing with water before filling with autoclaved media.

**Table 1:** Initial and final nutrient concentrations (mean±st.dev., n = 3) of JSM on three different cultivation design-units (MLHD, BPVD, SD and SUB).

Design	NO_3_^-1^	NH_4_^+^	DIN	PO_4_^-3^
	*μM*	*μM*	*μM*	*μM*
**Initial**				
1X	252.0	166.8	419.2	30.8
2X	502.0	329.2	831.6	60.4
4X	1008.0	667.2	1676.8	123.2
8X	2440.0	1652.8	4099.2	328
**Final: MLHD**				
1X	1.0±1.1	1.4±1.2	5.4±6.1	15.4±12.2
2X	21.3±29.7	0.6±0.5	22.0±30.4	4.9±4.3
4X	67.3±5.5	1.4±0.6	47.0±37.9	5.5±4.8
8X	302.52±98.42	88.9±78.6	392.7±178.0	37.3±16.1
**Final: BPVD**				
1X	1.4±0.5	1.5±0.4	3.0±0.9	19.4±7.3
2X	57.8±61.8	3.4±0.4	61.5±61.8	29.8±2.6
4X	615.1±206.3	7.4±0.6	625.7±207.0	66.8±22.4
8X	1861.6±347.6	214.3±57.4	2081.7±292.6	50.7±1.4
**Final: SD**				
1X	0.3±0.0	0.3±0.0	0.6±0.1	2.1±0.6
2X	0.4±0.1	0.5±0.2	0.9±0.2	2.7±0.2
4X	87.8±15.5	2.3±0.8	90.8±14.3	11.8±1.2
8X	271.8±89.0	58.1±13.9	330.4±76.6	32.5±8.6
**Final: SUB**				
1X	0.57±0.43	0.5±0.3	1.1±0.8	1.1±1.6
2X	1.69±0.56	2.1±0.6	3.9±1.2	5.7±1.9
4X	2.48±0.35	4.3±1.1	55.7±85.8	12.9±17.5
8X	7.42±1.61	497.7±40.3	508.2±39.1	2.8±0.2

A randomized framework (S1 Fig) was employed to test for physiological responses to culture factors, nutrient concentrations and culture platform design differences.

The *Ulva compressa* (UC-CC-ST1) starter biomass used in this study was in unialgal culture at the Marine Biotechnology Laboratory of the University of Connecticut at Stamford. The strain of *U*. *compressa* was originally collected from Bridgeport, CT. This *Ulva compressa* was induced to release spores and germinate. Individual germlings were transferred to von Stosch enriched (VSE) seawater medium and maintained at 20°C, 60 μmol photon m^-2^ s^-1^ light and day neutral conditions (12:12, L:D). This *Ulva* strain was genetically typed per the techniques of Mao et al. [49]. At SIO, the UCONN-supplied biomass was conditioned for two weeks in the carboys using normal seawater-based von Stosch media under ca. 75 μmol photon m^-2^ s^-1^ illumination prior to commencement of the experiments.

At the termination of the biomass culture runs (15-days), all of the cultured biomass from each treatment was harvested and weighed after blotting on paper towels, then dried in a low temperature oven (60°C). Dry weight was estimated using the formula [50]: Dry Biomass Productivity (DBP) = (Wtf * (HWti/Wti)/A)/d (g m^-2^ d^-1^), where Wtf is the final dry weight and HWti/Wti is the wet biomass correction factor (i.e., highest wet biomass weight ÷ /initial mean weight of sample for each design grouping). The initial weights for each grouping showed differences of ± 0.01 g); A = m^2^ (0.004 m^2^, normalized area of sample; 6.35 cm^2^); d = number of cultivation days (= 15). Daily growth rate/growth rate (as %GR d^-1^) was calculated using the difference of the final and initial weight divided by the number of days in the culture and expressed as % [(Wt/Wo)^1/t^-1]*100. [50–51]. Each experimental culture container was stocked with approximately 12 g of pre-weighed seaweed biomass. For this study, we conducted the following numbers of culture trials: MLHD and SD, n_t_ = 3; BPVD, n_t_ = 6; SUB, n_t_ = 1 trials. Replicate number was three (n_r_ = 3). The high number of trials in each treatment minimized the effect of lost very small pieces of seaweed biomass which inadvertently occurred during the stocking of the culture containers, and at harvest; (see photo illustration in Fig 1).

Nutrient analyses were performed by the Scripps Institution of Oceanography Ocean Data Facility, on a Seal Analytical continuous-flow AutoAnalyzer-3 (AA3) using published methods [52–54].

Steady state kinetics of nutrient uptake and seaweed growth was employed [55–56]. The uptake kinetics are dependent upon the rate of the nutrient uptake in relation to the nutrient concentration of the medium, as expressed by the Michaelis-Menten equation: *V* = *V*_max_ x C/K+C, where C is the nutrient concentration in the medium; *V* and *V*_max_ are the specific and maximum specific uptake rates; and K is the half saturation constant, equal to the nutrient concentration, when *V* = *V*_max_/2, μmol.

Pigments (Chl) were extracted from the wet harvested seaweed biomass using a glass mortar and pestle in 90% acetone. The spectra of extracted pigments were determined by a Varian Cary 100 Spectrophotometer. chl *a*, chl *b* and carotene were calculated as published previously [57]. For organic carbon and nitrogen analyses, harvested biomass was first acidified to remove carbonates, and dried for 24 hours at 50°C. Samples were sent to the UC Santa Barbara MSI Analytical Laboratory and analyzed using a Control Equipment Corporation, CEC 440HA CHN analyzer.

Normality of data was tested using the Shapiro-Wilkinson test and the Brown-Forsythe test for variance equality. The interactive effects of the various culture designs and the varying JSM concentrations on the growth rate were verified through one-way and two-way ANOVA analyses. These were followed by applying the Holm-Sidak method for pairwise multiple comparison (at a significance level of 0.05). All statistical analysis was performed using SigmaPlot (Version 2016). A randomized two-factorial experimental design was chosen to test the three-duplicated drip irrigation platform designs and the four fertilizer concentration effects, and their interactions with growth performance and protein production in each treatment in comparison to the triplicated fully submerged control treatments.

## Results

**Fig 2:**
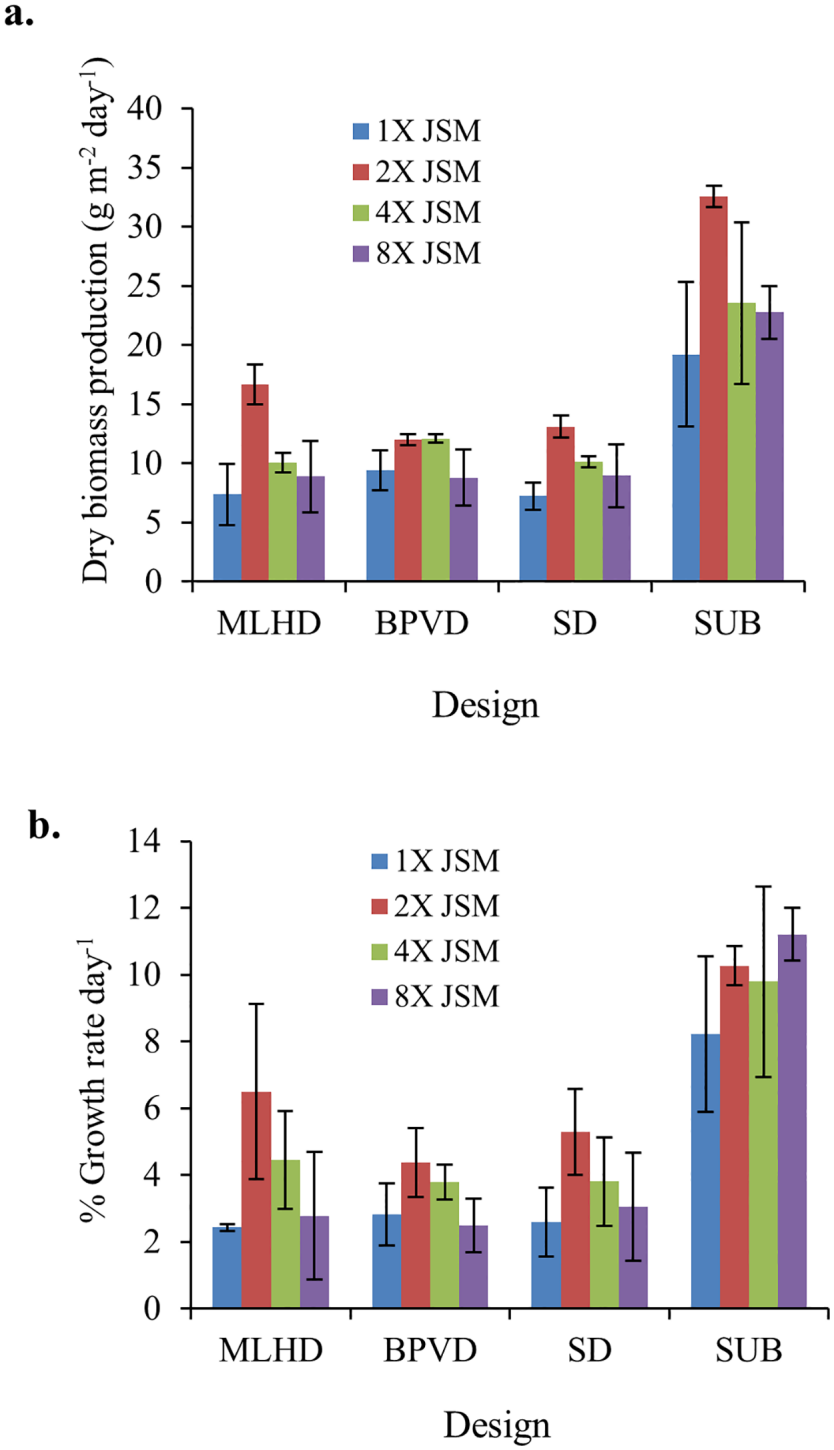
**a)** Mean biomass production per day of *Ulva compressa* grown at different JSM concentrations (1X-8X) on three different cultivation drip-irrigated system cultivations for 15 days in culture; (mean ± st.dev., n = 3 replicates; note: each replicate is an average of 3 trials); **b**) % Growth rate per day of *Ulva compressa* grown at different JSM concentrations (mean ± st.dev., n = 3) on three different cultivation spray system cultivation for 15 days.

Comparing the mean daily dry biomass productivity from the three drip irrigation platforms to the submerged control platform, showed that the submerged control platform consistently maintained the highest biomass productivity (range = from 20–30 g m^-2^ d^-1^), whereas biomass productivity for the three drip-irrigated platforms ranged from 5–18 g m^-2^ d^-1^, (Fig 2).

Significant biomass productivity differences were observed (p<0.001) within the JSM commercial fertilizer treatments and the three different drip culture platform designs. However, this was not the case for biomass productivity between JSM treatments, and for between culture platform design interactions (p = 0.492; Table 2). Within the JSM treatments, biomass productivity ranged considerably between the 1X and 2X, and 2X and 8X fertilizer concentration levels (Table 2). Within the design treatments, MLHD (p<0.001), and BPVD (p <0.001) showed significantly lower biomass productivity when compared to the submerged platform controls (SUB).

**Table 2:** Biomass data passed normality (Shapiro-Wilk; P = 0.087) after log transformation and equal variance tests (Brown-Forsythe; p = 0.682). Two-way Analysis of variance of the JSM fertilizer concentrations (1X, 2X, 4 X and 8X) and cultivation design (MLHD, BPVD, SD, SUB) on the daily growth rate of *U*. *compressa*. *The mean difference is significant at the 0.05 level.

Source of Variation	Sum of Squares	df	Mean Square	F	p
JSM*	0.407	3	0.136	15.321	<0.001
Design*	1.387	3	0.462	52.267	<0.001
JSM x Design	0.076	9	0.008	0.957	0.492
Residual	0.283	32	0.009		
Total	2.153	47	0.046		

Testing the different JSM fertilizer concentration treatments among culture platform designs indicated that growth rates (= rate of change in mean dry biomass weight per day) of *Ulva* in the MLHD (p = 0.001), BPVD (p = 0.001), SD (p<0.001) drip-irrigation designs were significantly lower than that of the totally submerged controls. The growth rate of *Ulva* with JSM treatments among MLHD designs indicates significant increases for the 2X treatments (p<0.001) and the 4X treatments (p = 0.004), when compared with 1X and 8X concentration treatments, respectively. No significant growth increases were observed within the BPVD designs, namely, 4X with 1X (p = 0.481) and 8X (p = 0.310), and with 2X with 8X (p = 0.289) concentration treatments. For the SD and SUB designs, significant increase in growth rate can only be found for 1X (p = 0.01) when compared with 2X concentration treatments.

Significant differences in the growth rate among the different concentrations of JSM was observed after allowing for effects of differences in the cultivation platforms and the different JSM dose interactions (p≤0.001; Table 3). To isolate which group(s) differed from the others, a univariate multiple pairwise comparison procedure was employed. This analysis showed the highest growth rate of dry biomass/day was obtained using the 2X JSM treatment, with significant variation at 1X (p<0.001), followed by 8X JSM (p<0.001). The different JSM concentrations and their interaction with the drip platform design type as a factor, showed no significant effect (p = 0.492) on the mean daily dry biomass production, indicating that the drip platform design does not depend on the nutrient levels to either increase or decrease seaweed growth rate and productivity.

**Table 3:** Pairwise comparison using Holm-Sidak method comparing interaction of effects of design (MLHD, BPVD, SD, SUB) and JSM fertilizer concentrations (1X, 2X, 4 X and 8X). Overall significance level = 0.05. *The mean difference is significant at the 0.05 level.

*Comparison*	Diff of Means	t	p
*Factor*: *JSM*			
1X vs. 2X	0.248*	6.446	<0.001
2X vs. 8X	0.185*	4.806	<0.001
1X vs. 4X	0.136*	3.539	0.005
2X vs. 4X	0.112*	2.907	0.020
4X vs. 8X	0.073	1.899	0.129
1X vs. 8X	0.063	1.640	0.111
*Factor*: *Design*			
MLHD vs. SUB	0.411*	10.710	<0.001
BPVD vs. SUB	0.389*	10.138	<0.001
BPVD vs. SUB	0.374*	9.729	<0.001
MLHD vs. BPVD	0.038	0.980	0.705
MLHD vs. SD	0.022	0.572	0.816
BPVD vs. SD	0.016	0.409	0.685
*Factor*: *JSM within 1X*			
MLHD vs. SUB	0.421*	5.480	<0.001
SD vs. SUB	0.411*	5.346	<0.001
BPVD vs. SUB	0.297*	3.867	0.002
MLHD vs. SD	0.124	1.613	0.310
BPVD vs. SD	0.114	1.479	0.276
MLHD vs. SD	0.010	0.134	0.894
*Factor*: *JSM within 2X*			
BPVD vs. SUB	0.433*	5.086	<0.001
SD vs. SUB	0.396*	5.060	<0.001
MLHD vs. SUB	0.292*	4.038	0.001
MLHD vs. SUB	0.141	1.048	0.661
MLHD vs. SD	0.104	1.022	0.530
SD vs. BPVD	0.038	0.0256	0.980
*Factor*: *JSM within 4X*			
MLHD vs. SUB	0.391*	5.086	<0.001
SD vs. SUB	0.389*	5.060	<0.001
BPVD vs. SUB	0.310*	4.038	0.001
MLHD vs. BPVD	0.081	1.048	0.661
BPVD vs. SD	0.079	1.022	0.530
MLHD vs. SD	0.002	0.026	0.980
*Factor*: *JSM within 8X*			
BPVD vs. SUB	0.454*	5.915	<0.001
MLHD vs. SUB	0.454*	5.913	<0.001
SD vs. SUB	0.450*	5.863	<0.001
BPVD vs. SD	0.004	0.053	1.000
MLHD vs. SD	0.004	0.050	0.998
MLHD vs. BPVD	0.000	0.002	0.998
*Factor*: *Design within MLHD*			
1X vs. 2X	0.375*	4.879	<0.001
2X vs. 8X	0.287*	3.742	0.004
2X vs. 4X	0.218*	2.843	0.031
1X vs. 4X	0.156	2.036	0.143
1X vs. 8X	0.087	1.137	0.458
4X vs. 8X	0.069	0.9000	0.375
*Factor*: *Design within BPVD*			
4X vs. 8X	0.150*	1.950	0.310
2X vs. 8X	0.146*	1.904	0.289
1X vs. 4X	0.113*	1.471	0.481
1X vs. 2X	0.109*	1.425	0.416
1X vs. 8X	0.037	0.479	0.867
2X vs. 4X	0.004	0.046	0.963
*Factor*: *Design within SD*			
1X vs. 2X	0.260*	3.392	0.011
2X vs. 8X	0.180	2.339	0.122
1X vs. 4X	0.148	1.928	0.228
2X vs. 4X	0.112	1.464	0.392
4X vs. 8X	0.081	1.053	0.510
1X vs. 8X	0.067	0.875	0.388
*Factor*: *Design within SUB*			
1X vs. 2X	0.246*	3.197	0.019
1X vs. 4X	0.126	1.642	0.443
2X vs. 8X	0.125	1.627	0.382
1X vs. 8X	0.121	1.569	0.333
2X vs. 4X	0.119	1.554	0.243
4X vs. 8X	0.006	0.073	0.942

The data for nutrient uptake rates were estimated using the Michaelis-Menten equation at the different JSM fertilizer concentrations (Table 4). *V*_max_*/K*_m_ for the BPVD drip-irrigated platform design showed better DIN uptake (NO_3_^-1^ + NH_4_^+^) efficiency when compared to the other two drip-irrigated platform designs, while the submerged control platform (SUB) showed the lowest uptake rate for DIN, (which is peculiar and unexplainable considering the highest biomass productivity was observed for the submerged controls (e.g., 20–30 g m^-2^ d^-1^). *Ulva* grown on each of the different drip irrigation- platforms (SD, MLHD and BPVD) exhibited higher overall nutrient uptake than did *Ulva* biomass grown using the submerged platform controls (SUB). This was found to also be true for nitrate, ammonium, and phosphate nutrients. *Ulva* cultured on platforms MLHD and SD had higher preference for nitrate uptake, while BPVD tended to have more preference for uptake of ammonium and phosphate. As shown in the kinetic parameters (Table 4), *Ulva’s* maximum uptake rate and half-saturation values for nitrate on the MLHD and SD platforms were similar and closely related to each other (Table 4). Employing linear regression analysis, BPVD showed significantly higher ammonium (p<0.001) uptake rates than the SUB controls (p = 0.034). Uptake rates for PO_4_ were significantly higher (p = 0.008) for each of the three drip-irrigation platforms when compared to the submerged (SUB) controls (p = 0.045; Table 4).

**Table 4:** Kinetic parameters *V*_max_ of *U*. *compressa*. A small K*m* for BPVD indicates the enzyme requires only a small amount of substrate to become saturated. *V*_max_ represented as the maximum reaction velocity. Standard error and significance (alpha = 0.05) of the linear regression analysis of the linearized form of the Michaelis-Menten equation (Lineweaver-Burk double reciprocal plot).

Design	Kinetic	Parameters		Linear	Regression	
	*V*_*max*_	*K*_*m*_	*V*_*max*_*/K*_*m*_	*R*^*2*^	*std*. *error*	*p*
	**NO**_**3**_^**-1**^					
MLHD	93.46	3.31	0.028	0.87	0.026	0.068
BPVD	39.37	0.62	0.064	0.89	0.011	0.059
SD	99.01	3.48	0.028	0.89	0.024	0.056
SUB	26.95	7.58	0.004	0.94	0.141	0.033
	**NH**_**4**_^**+**^					
MLHD	83.33	3.00	0.028	0.89	0.037	0.059
BPVD	335.57	7.76	0.043	0.99	0.001	<0.001
SD	81.33	2.91	0.028	0.88	0.038	0.060
SUB	10.12	2.77	0.004	0.93	0.213	0.034
	**DIN**					
MLHD	183.49	6.60	0.028	0.88	0.015	0.062
BPVD	221.73	4.58	0.048	0.99	0.003	0.006
SD	180.18	6.38	0.028	0.89	0.014	0.057
SUB	33.11	9.19	0.004	0.94	0.084	0.032
	**PO**_**4**_^**-3**^					
MLHD	4.50	0.34	0.013	0.99	0.102	0.004
BPVD	5.15	0.33	0.015	0.99	0.126	0.008
SD	15.29	0.59	0.026	0.90	0.199	0.049
SUB	2.21	0.64	0.003	0.91	1.438	0.045

**Fig 3:**
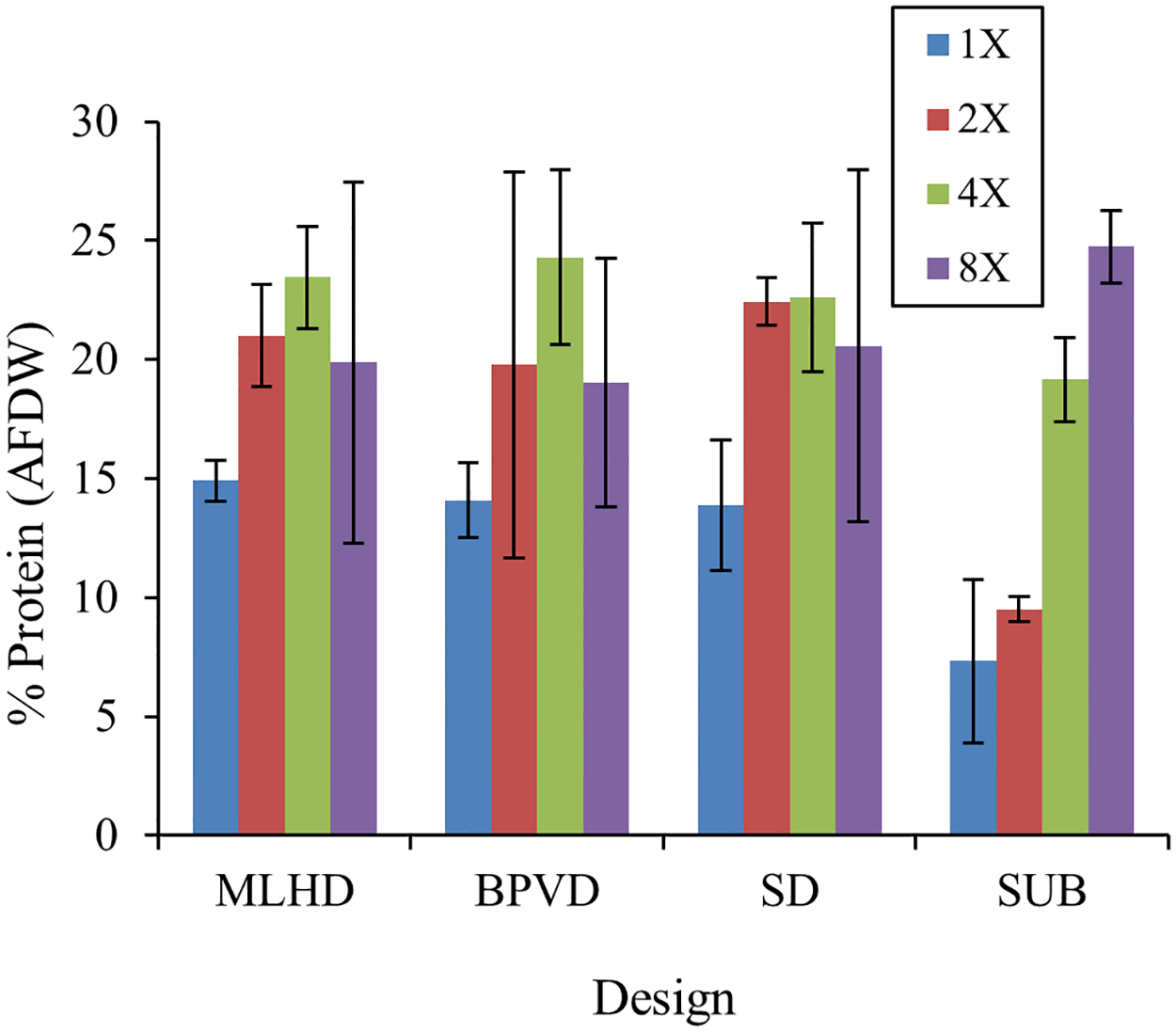
% Protein content of *U*. *compressa* grown for 15 days in the different cultivation systems at different concentration of JSM media (mean ± st.dev., n = 3).

The high DIN uptake rate observed for the BPVD platform design corresponds to a considerably higher protein content of the harvested biomass for that platform (Fig 3). For the nutrient treatments, the 1X fertilizer concentration treatment in the SUB control group showed significantly lower protein content than did the 2X (p = 0.020), 4X (p<0.001) and 8X (p<0.001) concentrations for the three drip irrigation platforms. This result indicates that protein content of the biomass produced using the three drip culture platforms was equal or higher than that of the biomass cultured using the SUB control. The BPVD platform showed appreciable growth performance and better protein production when compared with the MLHD platform, when fertilized with 2X media concentration, where the absolute quantity of protein produced in the MLHD platform was not significantly different from that of either the PBVD or SD platforms. However, the BPVD platform did perform somewhat better than the MLHD and SD platforms (for protein content), in that this platform yielded the maximum protein content observed from any of the experimental treatments (~27% d.w.) when the biomass was cultured using the 2X JSM media fertilizer concentration.

## Discussion and conclusions

Our results showed that both the sloped (SD) drip culture platform design, and the multi-level horizontal design (MLHD) produced significantly higher rates of NO_3_ uptake when compared to the totally submerged controls (SUB). Additionally, the bag-pouch vertical drip culture platform design (BPVD) produced the highest uptake rates of NH_4_^+^ and PO_4_^-3^ when compared to the SUB controls. These results support a physiological adaptation mechanism in the drip irrigated groups expressed as increased rates of liquid and gas mass transfer through relatively thinned cellular diffusive boundary layers mediated by the continual and repeated “pelting” of steady streams of nutrient-rich media falling onto and thence off of the thalli surfaces. This mechanism does not apply for the totally submerged controls where water movement past the seaweed thalli is much, much reduced compared to the fast-paced drip irrigation.

Prior published literature supports this conclusion, wherein the authors report increased capacity for nutrient uptake and higher photosynthetic rates for high littoral species measured in the field, as compared to species living further down (more submerged) in the littoral zone that are, overall, less exposed to air during their life cycles [58–60].

Another illustrative field study of this phenomenon [61] was conducted at the Stanford University, Hopkins Marine Station in California, where found that for a number of mid- and high-intertidal species (, i.e., *Iridaea flaccida*, *Porphyra perforate*, *Fucus distichus*, *and Endocladia muricata*), photosynthetic rates–(measured as mgCO_2_ dm^-2^ hr^-1^) increased over a range of 1.6x to 6.6x when the tested seaweeds were exposed to air and allowed to desiccate, then was measured for the same colonies when they were totally submerged by the incoming tide. Also, and from the same study two low intertidal species, *Ulva expansa* and *Prionitis lanceolota*, showed reduced photosynthetic rates in air as compared to when they were totally submerged.

Yet another study [40] looked at two New England *Porphyra* species for their ability to withstand desiccation stress. This study found that the sublittoral species *Porphyra amplissima* increased its uptake of nitrate following severe desiccation stress, whereas the rate of nitrate uptake for a eulittoral species *Porphyra umbilicalis* was not significantly affected following prolonged periods of desiccation. Other supporting studies [62– 63] present evidence of increased growth and photosynthetic rates for tank cultured intertidal species of the red seaweed genus *Gracilaria* (*G*. *ferox* and *G*. *tikvihae)*.

A comparative drip-irrigation study to ours (except that it was conducted out-of-doors under full sun in Israel) employed drip-irrigation of *Ulva lactuca* samples held on ceramic plates placed side-by-side on a cement pad at a slight upward-tilted angle facing the sun [17]. The authors reported growth rates of 11.8% day^-1^ and the biomass productivities of 171 g (f. wt.) m^-2^ day^-1^, which were somewhat higher than in our study (i.e., ~11% day^-1^ growth and ~ 150 g (f.wt.) m^-2^ day^-1^ biomass productivity). Given that our study was conducted indoors using fluorescent lighting (and theirs out-doors under much higher light intensities), the reduced light exposure of our study could certainly have accounted for the observed reduction in biomass productivities, as could inherent growth and adaptation differences between *Ulva* species.

It is also well documented in literature that water motion is important for uptake of dissolved nutrients (e.g. ionic compounds) into seaweed cells from the surrounding media via active transport and passive diffusion [60, 64–66]. Thus, the increased water motion experienced by intertidal species can enhance mass transfer rates for gases and nutrients, as is the case for our drip irrigation methodology, where the water motion onto and off of the seaweed thalli (breaking down diffusive boundary layers) can greatly increase mass transfer rates [60].

Pigment content of the harvested biomass Chl *a*, Chl *b* and carotene (S2 Table) was highest for the biomass grown using the SD culture platform and enhanced in that treatment when compared to the pigment content in the fully-submerged controls. These results indicate that pigments, *per se*, are not necessarily a controlling factor directly affecting the biomass production in *U*. *compressa*–at least not under moderate levels of illumination applied in this study [63].

Although light levels ranged from 120–250 μmol photon m^-2^ s^-1^ with a mean value of 179.5 ± 46.1 (S1 Table), literature reports [67–68] indicate *Ulva sp*. saturate growth and photosynthesis between about 120–150 μmol photon m^-2^ s^-1^ and are not inhibited at our highest irradiances. Therefore, we are confident our light levels saturated but did not photo-inhibit growth. Furthermore, SUB 2x had the highest overall growth rate but at an irradiance of 140 μmol photon m^-2^ s^-1^, while the other SUB treatments had irradiances > 200 μmol photon m^-2^ s^-1^, while the 2x MLHD had the highest growth rate for MLHD treatments at an irradiance of 118 and all other MLHD treatments had irradiances >175 μmol photon m^-2^ s^-1^. Given these observations and based on other reports in the literature that carried-out light-dependent experiments, we are confident that all irradiances used in our study were saturating for growth.

Our results also showed an increase in the *V*_max_*/K*_m_ ratio for the BPVD support platform, which also suggests enhanced efficiency in DIN uptake (NO_3_^-1^ + NH_4_^+^). And again, our results are supported by results obtained in another field study [39, 69], which showed enhanced uptake of nitrate and nitrite following extensive desiccation stress for two inter-tidal seaweed species from British Columbia, Canada (*Fucus distichus* and *Pelvetiopsis limitata*).

The pH of the culture media in the reservoirs (i.e., MLHD, BPVD, SD) decreased by 0.5 to 1.0 pH units over the course of the growth experiments. This was in contrast with the pH in the submerged platform reservoir, which maintained relatively basic pH (increased pH) over the entire course of the experiment (S1 Table). Increased pH has been observed to enhance ammonia uptake, whereas inorganic carbon availability in slow moving water increases the pH, causing relative proportions of CO_2_ and HCO_3_^-^ to decrease [70]. At pH 9.0 or above, 90% of contributing ions are that of carbonate, while an increase in HCO_3_^-^ is found with the pH range of 7.9–8.2.

Another study showed that *Ulva lactuca* did not utilize carbonate ions efficiently, and a high concentration of carbonate ions in the seaweed thalli led to carbon limitation, which, in-turn, led to reduced photosynthesis as indicated by reduced chlorophyll concentrations (S2 Table) and reduced growth rate [71]. Thus, it could be hypothesized that pH stress in our study could at least partially account for the reduced growth rates of the drip-irrigated platforms over that measured for the control group (SUB). However more research would need to be conducted to further test this hypothesis to uncover a probable mechanism of action.

Intertidal seaweeds are potentially susceptible to photo-inhibition of growth, since high light levels in the shallow inter-tidal has been correlated with reduced rates of photosynthesis [37, 72]. This is due to the general observation that the photochemical apparatus of most intertidal seaweeds operates to optimize photosynthesis at low light conditions associated with full immersion. Accordingly, these light-mediated physiological responses will need to be taken into consideration for appropriate species selection for drip irrigation culture in the proposed high-light greenhouse environments where species from the high intertidal littoral zone should be better adapted to the higher light levels than species adapted to growth and survival in the sublittoral or even the eulittoral (mid-littoral) zones.

Our data showed a moderate decrease in the mean biomass productivity with the different drip-irrigated configurations (e.g. range: 5–18 g m^-2^ d^-1^), although the highest productivity (18 g m^-2^ d^-1^), was essentially the same as the lower range of the submerged controls, namely 20 g m^-2^ d^-1^. However, we did measure an increase in total protein content for the drip-irrigated cohorts over that of the fully-submerged controls, indicating that the drip-irrigated seaweeds received sufficient water emersion and nutrient uptake to biosynthesize inorganic nutrients to organic cellular products.

Although mean biomass production for the drip-irrigated biomass was found to be somewhat lower than that for the fully submerged controls, the overall benefits of employing the drip irrigation methodology: e.g. 80% water savings, higher nutrient removal rates, nutrient conservation and recycling (Table 4); and the ability to use industrial flue gas for low-cost carbonation of seaweed photosynthesis, should out-weigh any minor reductions that might be experienced in overall biomass production at commercial scale.

This simple lab-study has demonstrated that drip-irrigated *Ulva* seaweed grown using a vertically-oriented culture platform can exhibit improved protein content of cultured biomass when provided excess macronutrients. The bag-pouch vertical support design (BPVD) represents a new platform design for culturing *Ulva* and other seaweed biomass to better utilize the vertical (volume) space and scattered light within a 3-dimensional, fully wetted greenhouse environment. Based on measured K_m_ values, the hanging BPVD required less energy to catalyze protein production within the plant thalli than did the other two drip-irrigated designs, and/or the fully submerged control designs, while producing higher protein content biomass than either of the other drip designs tested, and/or the fully submerged tank culture methodology

The protein content results found in this study for the drip irrigation platforms (~27% of dry weight matter) is double the values reported for *Ulva rigida* grown in offshore fish farm pens [73] and is similar to protein content of *Ulva* spp. grown in outdoor drip-irrigated culture in Israel using fish farm effluents [17, 35]. On a calculated basis, we estimate that it would require 4000 μM of DIN to produce 230 μg g^-1^ dry biomass/day of protein in an *Ulva* drip-culture greenhouse enclosed biomass production system.

*Ulva* is a promising candidate for remediating wastewater effluents, for example from fed fish aquaculture (e.g. Integrated Multi-Trophic Aquaculture (IMTA) practice), before effluents are released into natural receiving waters.

We suggest, that use of bag pouch design (BPVD) tested in this study as a model for a scaled-up commercial prototype system would allow for better use of vertical space within a semi-enclosed culture environment (a greenhouse-, and thus allowed for increased productivity on a given land-area basis, versus a one-level horizontal culture design. Due to the slim design and transparent plastic platform, the easy to construct and clean BPVD design also minimizes mutual light shading, which would improve overall biomass and areal productivity from a given land footprint area. The Multi-Level Horizontal design (MLHD) provided the highest growth rate observed when using 2X concentration of Jacks Special Media formulation (JSM), so it too could be considered as a viable candidate for trials in a scaled-up commercial prototype seaweed drip-irrigated seaweed production system.

Development of new markets for *Ulva* land-based cultivation could help promote new environmentally friendly seaweed culture businesses (e.g., development of seaweed-based shrimp aqua-feeds will increase the sustainability of the intensive shrimp culture industry and in for advancing inland aquaculture of shrimp).

This work provides baseline information that demonstrates that seaweed can produce a sustainable source of protein in land-based system with less water requirement for biomass production. Protein production from seaweeds using land-based approaches offers opportunity to support increasing demand for high protein source fishmeal and animal feeds in the coming decades of the 21st century.

## Supporting information

S1 Fig A randomized experimental design was employed to test the design and JSM concentration with growth performance of *Ulva* at alpha = 0.05. The study uses a two-factorial experiment to determine main effects and the interaction of these factors.
(TIF)

S1 Table Culture conditions of *Ulva compressa* grown in three different cultivation system designs. pH data was not obtained for the 2X treatment.
(DOCX)

S2 Table Chl *a*, Chl *b*, total Chl and total carotene (mean ± st dev, n = 3) grown in different cultivation design-units (MLHD, BPVD, SD and SUB). (DOCX)

S3 Table Water and AFDW content (ash-free dry weight) of *U*. *compressa* (mean ± st. dev, n = 3) grown on different cultivation design-units (MLHD, BPVD, SD and SUB) grown at different JSM concentrations. (DOCX)
