# Correction: Land-based drip-irrigated culture of *Ulva compressa*: the effect of culture platform design and nutrient concentration on biomass production and protein content

**DOI:** 10.1371/journal.pone.0201675

**Published:** 2018-07-27

**Authors:** Wilson Mendoza, Dominick Mendola, Jang Kim, Charles Yarish, Alyssa Velloze, B. Greg Mitchell

An affiliation is missing for the third author. In addition to the current affiliation information, Jang Kim is affiliated with: Department of Ecology & Evolutionary Biology and Department of Marine Sciences, Department of Marine Science, School of Natural Sciences, Incheon National University, 119 Academy-ro, Yeonsu-gu, Incheon 22012, Korea.
