# Supplementary figures and images for: Land-based drip-irrigated culture of Ulva compressa: The effect of culture platform design and nutrient concentration on biomass production and protein content
Source: PLoS One. 2018 Jun 27;13(6):e0199287. doi: 10.1371/journal.pone.0199287 (PMC6021086; doi:10.1371/journal.pone.0199287)

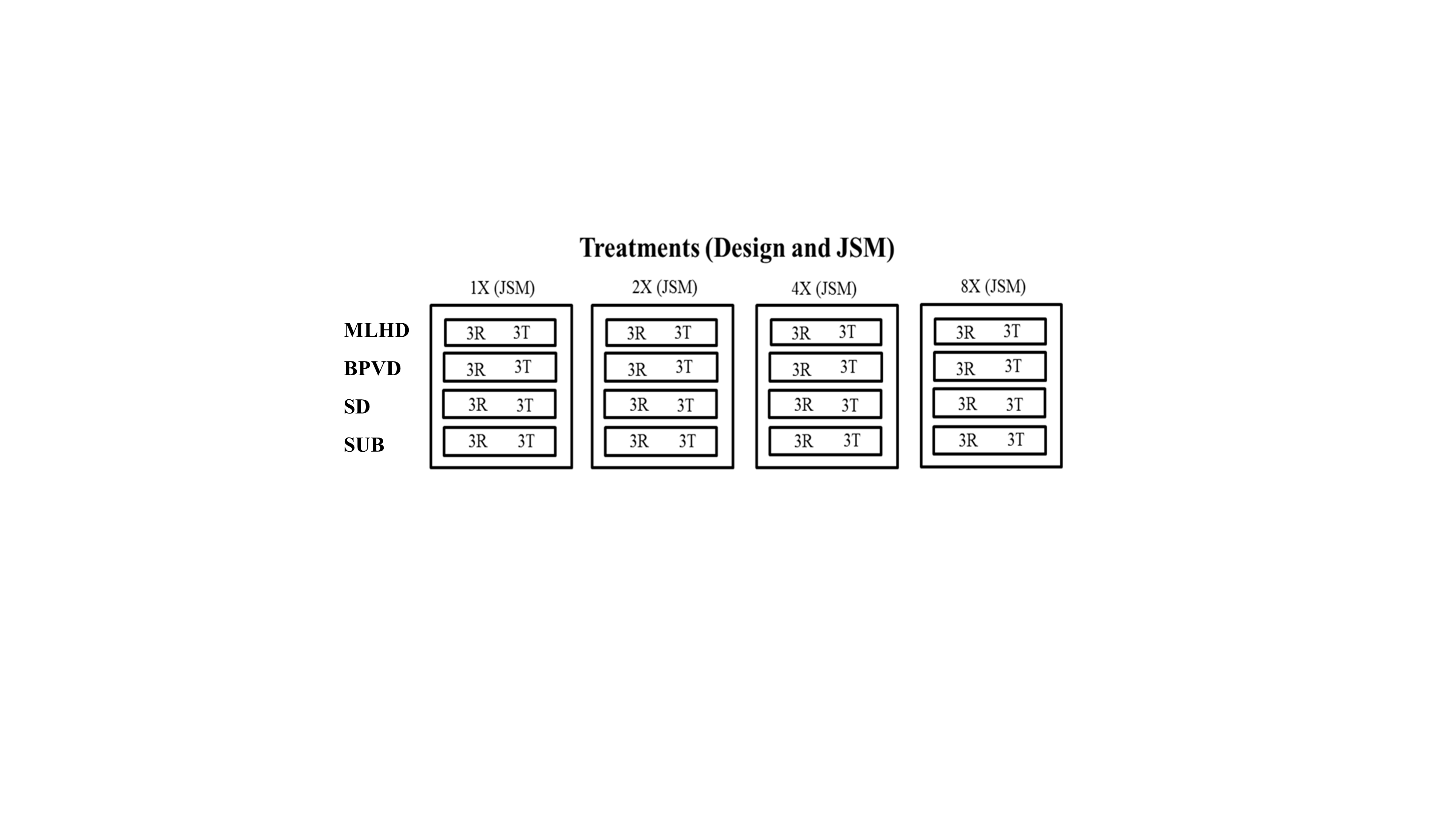

Supplement: S1 Fig — The study uses a two-factorial experiment to determine main effects and the interaction of these factors. (TIF) [file pone.0199287.s001.tif]
