# Supplementary material for: Land-based drip-irrigated culture of Ulva compressa: The effect of culture platform design and nutrient concentration on biomass production and protein content
Source: PLoS One. 2018 Jun 27;13(6):e0199287. doi: 10.1371/journal.pone.0199287 (PMC6021086; doi:10.1371/journal.pone.0199287)
Supplement: S1 Table — pH data was not obtained for the 2X treatment. (DOCX) [file pone.0199287.s002.docx]

**S1 Table**

|  |  | **pH** | **Temp (^o^C)** | **Light (µmol photon m^-2^ s^-1^)** |
| --- | --- | --- | --- | --- |
| **1X** | *MLHD* | 8.42±0.01 | 26.57±0.21 | 202.00±48.48 |
|  | *BPVD* | 8.47±0.03 | 25.90±0.40 | 157.73±16.83 |
|  | *SD* | 8.36±0.09 | 27.10±0.61 | 218.61±60.05 |
|  | *SUB* | 9.05±0.72 | 26.23±0.68 | 251.81±31.15 |
| **2X** | *MLHD* | - | 27.27±0.64 | 118.07±3.20 |
|  | *BPVD* | - | 26.30±0.44 | 136.51±27.86 |
|  | *SD* | - | 27.27±0.51 | 146.66±33.55 |
|  | *SUB* | - | 26.77±0.40 | 140.20±36.85 |
| **4X** | *MLHD* | 8.59±0.09 | 28.80±0.26 | 196.57±17.29 |
|  | *BPVD* | 8.57±0.13 | 26.63±0.15 | 164.27±26.89 |
|  | *SD* | 8.46±0.03 | 28.67±0.32 | 157.81±54.46 |
|  | *SUB* | 9.98±0.13 | 25.90±0.53 | 219.64±6.39 |
| **8X** | *MLHD* | 8.01±0.17 | 29.50±0.53 | 175.25±20.95 |
|  | *BPVD* | 7.81±0.12 | 27.47±0.57 | 171.56±5.53 |
|  | *SD* | 7.89±0.16 | 28.57±0.75 | 199.24±28.76 |
|  | *SUB* | 9.69±0.17 | 25.93±0.40 | 215.84±59.86 |
