# Supplementary material for: Land-based drip-irrigated culture of Ulva compressa: The effect of culture platform design and nutrient concentration on biomass production and protein content
Source: PLoS One. 2018 Jun 27;13(6):e0199287. doi: 10.1371/journal.pone.0199287 (PMC6021086; doi:10.1371/journal.pone.0199287)
Supplement: S2 Table — (DOCX) [file pone.0199287.s003.docx]

**S2 Table**

| **Design** | **Chl *a*** | **Chl *b*** | **Total Chl** | **Total Carotene** |
| --- | --- | --- | --- | --- |
|  | *mg g^-1^* | *mg g^-1^* | *mg g^-1^* | *ug g^-1^* |
| **1X** |  |  |  |  |
| *MLHD* | 0.40±0.29 | 0.39±0.39 | 0.79±0.58 | 0.76±0.38 |
| *BPVD* | 0.37±0.34 | 0.35±0.33 | 0.72±0.67 | 0.76±0.30 |
| *SD* | 0.67±0.38 | 0.65±0.31 | 1.31±0.69 | 0.78±0.25 |
| *SUB* | 0.13±0.12 | 0.14±0.14 | 0.27±0.26 | 0.40±0.23 |
| **2X** |  |  |  |  |
| *MLHD* | 0.24±0.19 | 0.23±0.18 | 0.48±0.37 | 2.59±1.52 |
| *BPVD* | 0.13±0.05 | 0.12±0.04 | 0.25±0.09 | 1.56±0.41 |
| *SD* | 0.29±0.11 | 0.27±0.10 | 0.56±0.21 | 1.96±0.39 |
| *SUB* | 0.14±0.07 | 0.13±0.06 | 0.27±0.13 | 3.14±1.81 |
| **4X** |  |  |  |  |
| *MLHD* | 0.77±0.24 | 0.76±0.27 | 1.53±0.51 | 1.64±0.48 |
| *BPVD* | 0.93±0.02 | 0.86±0.03 | 1.79±0.06 | 1.39±0.39 |
| *SD* | 1.01±0.38 | 0.90±0.36 | 1.91±0.74 | 1.20±0.12 |
| *SUB* | 0.49±0.15 | 0.49±0.15 | 0.98±0.30 | 0.52±0.06 |
| **8X** |  |  |  |  |
| *MLHD* | 0.07±0.09 | 0.06±0.08 | 0.13±0.17 | 0.34±0.51 |
| *BPVD* | 0.64±0.19 | 0.60±0.17 | 1.24±0.36 | 0.66±0.49 |
| *SD* | 0.90±0.43 | 0.83±0.40 | 1.73±0.83 | 0.80±0.56 |
| *SUB* | 0.74±0.08 | 0.66±0.09 | 1.40±0.17 | 1.58±0.66 |
