# Supplementary material for: Land-based drip-irrigated culture of Ulva compressa: The effect of culture platform design and nutrient concentration on biomass production and protein content
Source: PLoS One. 2018 Jun 27;13(6):e0199287. doi: 10.1371/journal.pone.0199287 (PMC6021086; doi:10.1371/journal.pone.0199287)
Supplement: S3 Table — (DOCX) [file pone.0199287.s004.docx]

**S3 Table**

| **Design** | **Water content** | **AFDW** |
| --- | --- | --- |
|  | *(%)* | *g m^-2^ d^-1^* |
| **1X** |  |  |
| MLHD | 74.53±4.95 | 5.72±0.14 |
| BPVD | 76.26±2.83 | 7.17±0.08 |
| SD | 77.87±1.35 | 5.92±0.05 |
| SUB | 77.75±0.87 | 14.05±0.36 |
| **2X** |  |  |
| MLHD | 77.37±1.19 | 13.18±0.02 |
| BPVD | 76.14±0.86 | 9.24±0.01 |
| SD | 78.92±0.94 | 10.59±0.04 |
| SUB | 73.39±0.96 | 26.43±0.02 |
| **4X** |  |  |
| MLHD | 79.67±1.37 | 7.93±0.02 |
| BPVD | 75.60±0.64 | 9.34±0.02 |
| SD | 80.54±0.84 | 7.89±0.00 |
| SUB | 75.77±1.06 | 17.86±0.09 |
| **8X** |  |  |
| MLHD | 81.17±2.65 | 6.98±.10 |
| BPVD | 75.34±5.32 | 7.22±0.10 |
| SD | 78.62±3.18 | 7.85±0.08 |
| SUB | 80.06±0.83 | 17.16±0.04 |
